# Influence of donor liver telomere and G-tail on clinical outcome after living donor liver transplantation

**DOI:** 10.1371/journal.pone.0213462

**Published:** 2019-03-07

**Authors:** Biou Liu, Kumiko Anno, Tsuyoshi Kobayashi, Jinlian Piao, Hidetoshi Tahara, Hideki Ohdan

**Affiliations:** 1 Department of Gastroenterological and Transplant Surgery, Graduate School of Biomedical and Health Sciences, Hiroshima University, Hiroshima, Japan; 2 Department of Cellular and Molecular Biology, Graduate School of Pharmaceutical Sciences, Hiroshima University, Hiroshima, Japan; Istituto Mediterraneo per i Trapianti e Terapie ad Alta Specializzazione, ITALY

## Abstract

It has been reported that donor age affects patient outcomes after liver transplantation, and that telomere length is associated with age. However, to our knowledge, the impact of donor age and donor liver telomere length in liver transplantation has not been well investigated. This study aimed to clarify the influence of the length of telomere and G-tail from donor livers on the outcomes of living donors and recipients after living donor liver transplantation. The length of telomere and G-tail derived from blood samples and liver tissues of 55 living donors, measured using the hybridization protection assay. The length of telomeres from blood samples was inversely correlated with ages, whereas G-tail length from blood samples and telomere and G-tail lengths from liver tissues were not correlated with ages. Age, telomere, and G-tail length from blood did not affect postoperative liver failure and early liver regeneration of donors. On the other hand, the longer the liver telomere, the poorer the liver regeneration tended to be, especially with significant difference in donor who underwent right hemihepatectomy. We found that the survival rate of recipients who received liver graft with longer telomeres was inferior to that of those who received liver graft with shorter ones. An elderly donor, longer liver telomere, and higher Model for End-Stage Liver Disease score were identified as independent risk factors for recipient survival after transplantation. In conclusion, telomere shortening in healthy liver does not correlate with age, whereas longer liver telomeres negatively influence donor liver regeneration and recipient survival after living donor liver transplantation. These results can direct future studies and investigations on telomere shortening in the clinical and experimental transplant setting.

## Introduction

Liver transplantation (LT) is a standard treatment for end-stage liver disease and liver malignancies. In a globally aging society, a declining pool for living donor liver transplantation (LDLT) and cadaver LT has become a critical issue. The possibility and safety of donations from marginal donors should be considered, particularly those of elderly and obese donors. It remains controversial whether donor age impairs recipient outcomes after LDLT [1]. However, the impact of donor age on the outcome of both donors and recipients after LDLT has not been studied.

For a long time, the liver was recognized as an organ that could regenerate; yet, the mechanism of liver regeneration is remains unclear. Eukaryotic organisms senesce as they get older, and organ function and regeneration ability decline. It has been reported that liver regeneration in elderly people and rats after hepatectomy slows down [2]. The residual capacity of hepatic function is thought to be correlated with liver regeneration. However, only a few studies have focused on the effects of aging liver tissues on liver regeneration and postoperative outcomes. Thus, it is necessary to clarify the relationship between liver regeneration and age.

Telomeres, double-stranded DNA containing repeat sequences of 5'-TTAGGG-3' at the ends of chromosomes, appear to be deeply involved in tissue regeneration, lifespan, and cell division [3, 4]. It has been reported that telomere length decreases as the time of cell division increases [3, 4]. According to the general theory, telomere length is inversely correlated with age. In addition, it has been reported that the telomeric 3’-overhang (G-tail) length is associated with a risk of cardiovascular events [5, 6]. However, the significance of telomere/G-tail length in LT has not been well studied.

We investigated the influence of telomere and G-tail length from donor blood and liver tissues on donor liver regeneration and recipient outcome after LDLT.

## Materials and methods

### Donors and recipients

Overall, 223 patients underwent LDLTs at Hiroshima University Hospital between 1991 and 2015. Blood and liver samples from 55 donors were collected at LDLT between 2010 and 2015. Written informed consent was obtained from all participants before surgery, in accordance with the Declaration of Helsinki. This study was approved by the Hiroshima University Institutional Review Board (HiM129-28). The procedure to protect the identity of the patients was subject to approval by the institutional review committee and met the guidelines of the responsible governmental authority.

LDLTs were performed at Hiroshima University Hospital, following the Japanese Liver Transplant Society guidelines. Donors were healthy adults who voluntarily applied to donate their liver. The size of the graft had to be more than 0.8% of the recipient's body weight [7]. Our donor/graft selection criteria, surgical procedures and immunosuppressant regimen are described in detail elsewhere [8]. Donors could not have malignant or infectious diseases, and the donor organs were limited to those from relatives whose relation to the recipient was within the third degree of consanguinity. Liver biopsies were performed in cases where an abnormality was found on computed tomography (CT). A less than mild fatty liver (< 10% of fat storage) was considered acceptable for transplantation [9].

### Perioperative measurements of hepatic morphology

Perioperative volumetric measurements of hepatic morphology were performed as previously described [7, 9]. Resection rates were used with the expected volume of the liver calculated on three-dimensional CT (3D-CT, version 3.1, GE Medical Systems, Milwaukee, WI, USA) and Zio 900 M (Zio Software, Tokyo, Japan) before surgery. We measured total liver volume, the future liver remnant (FLR) volume preoperatively, and liver remnant (LR) volume on day 7. The morphological regeneration rate of the liver on day 7 after liver resection (abbreviated as the early regeneration index: ERI) was calculated as [(VLR—VFLR)/VFLR] * 100, where VLR is the volume of the LR and VFLR is the volume of the FLR [2, 10].

### Postoperative outcomes

Donor outcomes included morbidity, post-hepatectomy-liver-failure (PHLF), and ERI. Recipient outcomes included morbidity and survival. The definition of PHLF was based on the International-Study-Group-of-Liver-Surgery (ISGLS), included the following: decrease in liver synthesis excretion, detoxification function, or symptoms of elevation of high bilirubin value; and international normalized ratio of prothrombin (PT-INR) value on day 5 (PT-INR-5), or later compared with the previous one if there was a high preoperative value, or a normal value that required “artificial supplements from the outside to maintain” [11, 12]. The standard value of each blood test refers to the reference value of each facility and laboratory.

### Quantification of telomere and G-tail length

Quantification of relative telomere length (RTL) and relative G-tail length (RGL), double-stranded and single-stranded, was performed using the telomere hybridization protection assay (HPA) method, as described previously [13, 14]. The HPA method represents telomere and G-tail length as luminescence signals (in relative light units [rlu]). DNA from whole blood and liver tissues were extracted using the phenol-chloroform method. For the telomere G-tail assay, 1 μg of non-denatured DNA was used to measure the G-tail, and 0.2 μg of denatured DNA (99°C for 10min) was used to measure the total telomere length. We also used control genomic DNA isolated from the HeLa cell line to normalize the luminescence. We took 1 μL from each sample tube and measured the DNA amount using NanoDrop (ND-2000; Thermo Fisher Scientific Inc., Waltham, MA, USA) to normalize the luminescence of each sample. Probes for acridinium ester (AE)-labeling of telomeres were supplied by Fujirebio (Tokyo, Japan). DNA was incubated with HPA probes for hybridization at 60°C for 20min, after then underwent hydrolysis at 60°C for 10min. The luminescence of AE relative to telomere length and G-tail length were measured using an EnVision multilabel reader (Perkin Elmer Japan Co Ltd).

### Statistical analysis

All statistical analyses were conducted using JMP 13 (SAS Institute Inc., Cary, NC, USA). The univariate analysis for continuous variables with normal distribution was compared using the Student's t-test. Continuous variables without a normal distribution were compared with Mann-Whitney's U test. A descriptive comparison was performed with the chi-squared test. Correlations between the presence of PHLF and continuous variables were expressed using Pearson's correlation coefficient. The area under the curve was calculated using the receiver operating characteristic (ROC) curve for the sensitivity and specificity of the value of the limit of telomere length. The difference between the two sides was considered statistically significant when the p-value was 0.05 or less. All relevant data are shown within Supporting Information files (S1 Table).

## Results

### Correlations between length of telomere and G-tail

**Fig 1:**
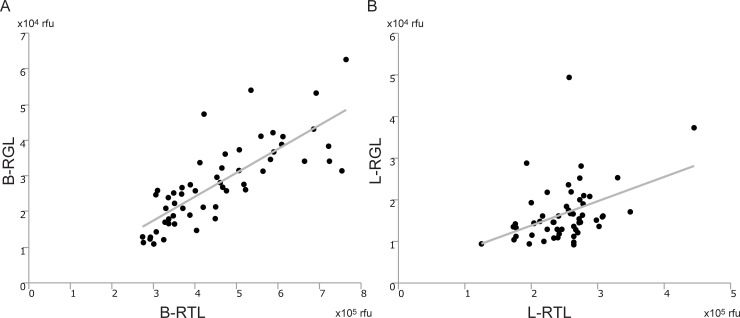
Correlation between telomere and G-tail length. (A) Correlation between B-RTL and B-RGL (y = 0.67 x—0.26, r^2^ = 0.622, p < 0.001). (B) Correlation between L-RTL and L-RGL (y = 0.58 x + 0.21, r^2^ = 0.175, p = 0.002). B-RTL; relative telomere length from blood, B-RGL; relative G-tail length from blood, L-RTL; relative telomere length from liver, L-RGL; relative G-tail length from liver.

Clinical characteristics of donors and recipients are shown in Table 1. First, we investigated the correlation between telomere and G-tail length from donor blood and liver tissue. Consistent with previous reports, relative telomere length from the blood samples (B-RTL) was significantly correlated with relative G-tail length from the blood samples (B-RGL) (Fig 1A). Additionally, relative telomere length from liver tissues (L-RTL) was also significantly correlated with relative G-tail length from liver tissues (L-RGL) (Fig 1B). Therefore, length of telomere was correlated with G-tail length in both blood and liver tissue.

**Table 1:** Clinical characteristics of patients.

Characteristics	
**Donors**	n = 55
Age, years	38.6 ± 12.4
Gender, Male	37 (67.3%)
Body mass index, kg/m^2^	22.2 ± 2.5
Type of Procedures	
Right hemihepatectomy	24 (43.6%)
Left hemihepatectomy	30 (54.5%)
Right posterior sectionectomy	1 (1.8%)
FLR, %	52.6 ± 12.1
FLR/SLV, %	54.3 ± 17.5
Operation time, min	452 ± 68
Blood loss, g	472 ± 279
Autologous transfusion	5 (9.1%)
PHLF	5 (9.1%)
ERI, %	36.7 ± 27.2
Hospital stay, days	14 ± 7
B-RTL, x10^5^ rfu	4.6 ± 1.4
L-RTL, x10^5^ rfu	2.5 ± 0.5
B-RGL, x10^4^ rfu	2.8 ± 1.2
L-RGL, x10^4^ rfu	1.7 ± 0.7
**Recipients**	n = 55
Age, years	57.4 ± 10.2
Gender, Male	27 (49.1%)
Body mass index, kg/m^2^	23.0 ± 3.5
Blood incompatibility	4 (7.3%)
hepatitis C virus infection	26 (47.3%)
MELD score	17.9 ± 7.7
Child-Pugh score	9.8 ± 1.9
GRWR	89.2 ± 16.3
Operation time, min	781 ± 146
Blood loss, g	4896 ± 3794

FLR; future liver remnant, SLV; standard liver volume, PHLF; posthepatectomy liver failure, ERI; early regeneration index, RTL; relative telomere length, RGL; relative G-tail length, MELD; Model for End-Stage Liver Disease, GRWR; graft-to-recipient weight ratio

### Differences of telomere/G-tail length between blood and liver

**Fig 2:**
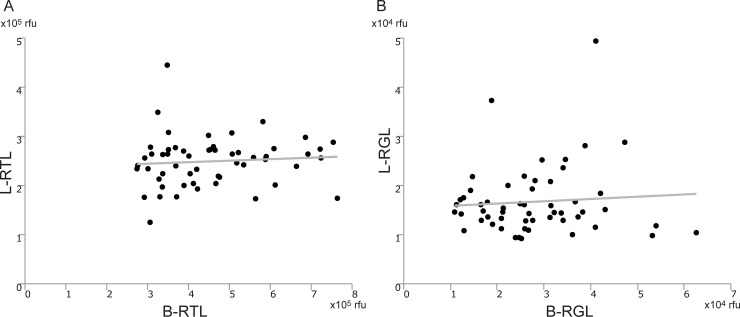
Correlation between telomere/G-tail length from blood and liver. (A) Correlation between B-RTL and L-RTL (y = 0.03 x + 2.35, r^2^ = 0.007, p = 0.552). (B) Correlation between B-RGL and L-RGL (y = 0.05 x + 1.54, r^2^ = 0.006, p = 0.583). B-RTL; relative telomere length from blood, L-RTL; relative telomere length from liver, B-RGL; relative G-tail length from blood, L-RGL; relative G-tail length from liver.

Next, we investigated the correlation between B-RTL and L-RTL, as well as that between B-RGL and L-RGL. L-RTL was not associated with B-RTL (Fig 2A), and B-RGL was not associated with L-RGL (Fig 2B). Consequently, length of telomere and G-tail was not correlated between blood and liver tissue.

### Correlation between telomere/G-tail length and age

**Fig 3:**
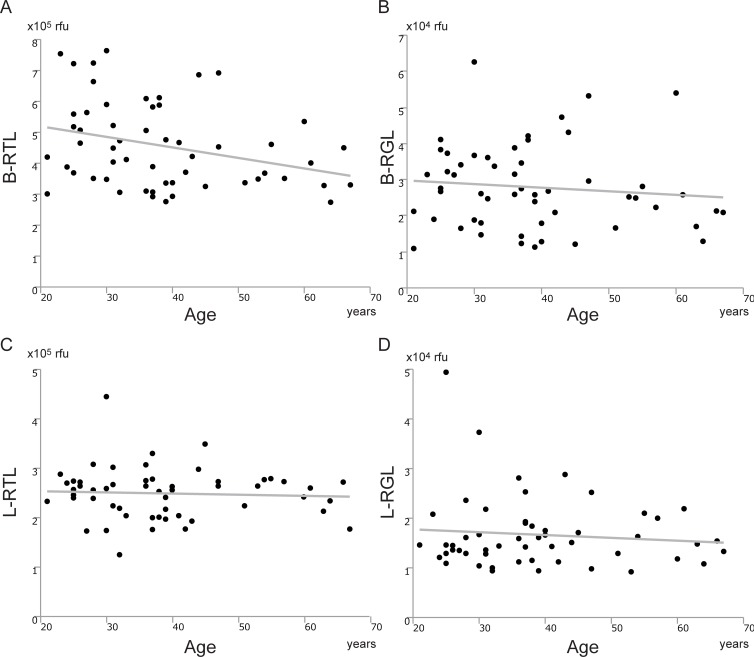
Correlation between telomere/G-tail length and age. (A) Correlation between B-RTL and age (y = -0.03 x + 5.87, r^2^ = 0.096, p = 0.021). (B) Correlation between B-RGL and age (y = -0.01 x + 3.17, r^2^ = 0.011, p = 0.437). (C) Correlation between L-RTL and age (y = -0.002 x + 2.58, r^2^ = 0.003, p = 0.679). (D) Correlation between L-RGL and age (y = -0.006 x + 1.89, r^2^ = 0.010, p = 0.468). B-RTL; relative telomere length from blood, B-RGL; relative G-tail length from blood, L-RTL; relative telomere length from liver, L-RGL; relative G-tail length from liver.

We investigated the correlation between telomere and G-tail length from blood and liver tissues and donor age. As shown in Fig 3, B-RTL was inversely correlated with age (Fig 3A); which is consistent with previous reports. B-RGL, L-RTL and L-RGL, however, were not correlated with age (Fig 3B–3D).

### Risk factors affecting donor PHLF

We reviewed preoperative characteristics of LDLT donors. Among 55 donors, 5 donors had PHLF, according to the ISGLS criteria. We were unable to identify any significant preoperative factors which affected PHLF (Table 2). Niether the future liver remnant nor the future liver remnant/standard liver volume were significant risk factors for PHLF. In addition, there was no significant difference between B-RTL, B-RGL, L-RTL, and L-RGL with and without PHLF.

**Table 2:** Clinical characteristics of liver donors according to PHLF.

	Univariate analysis		
	non-PHLF	PHLF	p value
Cases	n = 50	n = 5	
Age, years	39.4 ± 12.6	30.0 ± 6.7	1.107
<45	36	5	
≥45	14	0	
Gender			0.160
Male	32	5	
Female	18	0	
Total bilirubin level, mg/dl	0.92 ± 0.35	0.76 ± 0.32	0.324
aspartate aminotransferase, IU/l	19.8 ± 4.8	19.0 ± 2.1	0.705
alanine aminotransferase, IU/l	21.2 ± 9.3	21.0 ± 8.0	0.955
Albumin level, g/dl	4.7 ± 0.3	4.9 ± 0.3	0.360
ICG-R, %	7.3 ± 2.7	7.4 ± 3.3	0.979
PT-INR	1.03 ± 0.08	1.01 ± 0.10	0.605
Body mass index, kg/m^2^	22.1 ± 2.5	23.0 ± 2.6	0.483
Type of Procedures			0.236
Right hemihepatectomy	20	4	
Others	30	1	
FLR, %	53.3 ± 11.9	43.5 ± 10.7	0.084
FLR/SLV, %	54.8 ± 17.4	47.4 ± 20.2	0.376
Operation time, min	452 ± 71	476 ± 51	0.514
Blood loss, g	466 ± 276	575 ± 448	0.478
Autologous transfusion	5	0	1.000
Morbidity, Grade III or more	4	0	1.000
ERI, %	35.8 ± 27.6	45.1 ± 23.7	0.474
Hospital stay, days	14.1 ± 7.6	13.0 ± 4.4	0.753
B-RTL, x10^5^ rfu	4.5 ± 1.3	5.2 ± 1.7	0.321
L-RTL, x10^5^ rfu	2.5 ± 0.5	2.5 ± 0.6	0.847
B-RGL, x10^4^ rfu	2.7 ± 1.1	3.6 ± 1.6	0.095
L-RGL, x10^4^ rfu	1.7 ± 0.7	1.5 ± 0.6	0.602

ICG-R15; indocyanine green retention rate at 15 min, PT-INR; prothrombin time-international normalized ratio, FLR; future liver remnant, SLV; standard liver volume, ERI; early regeneration index, RTL; relative telomere length, RGL; relative G-tail length

### Risk factors that affect liver regeneration of donor livers

**Fig 4:**
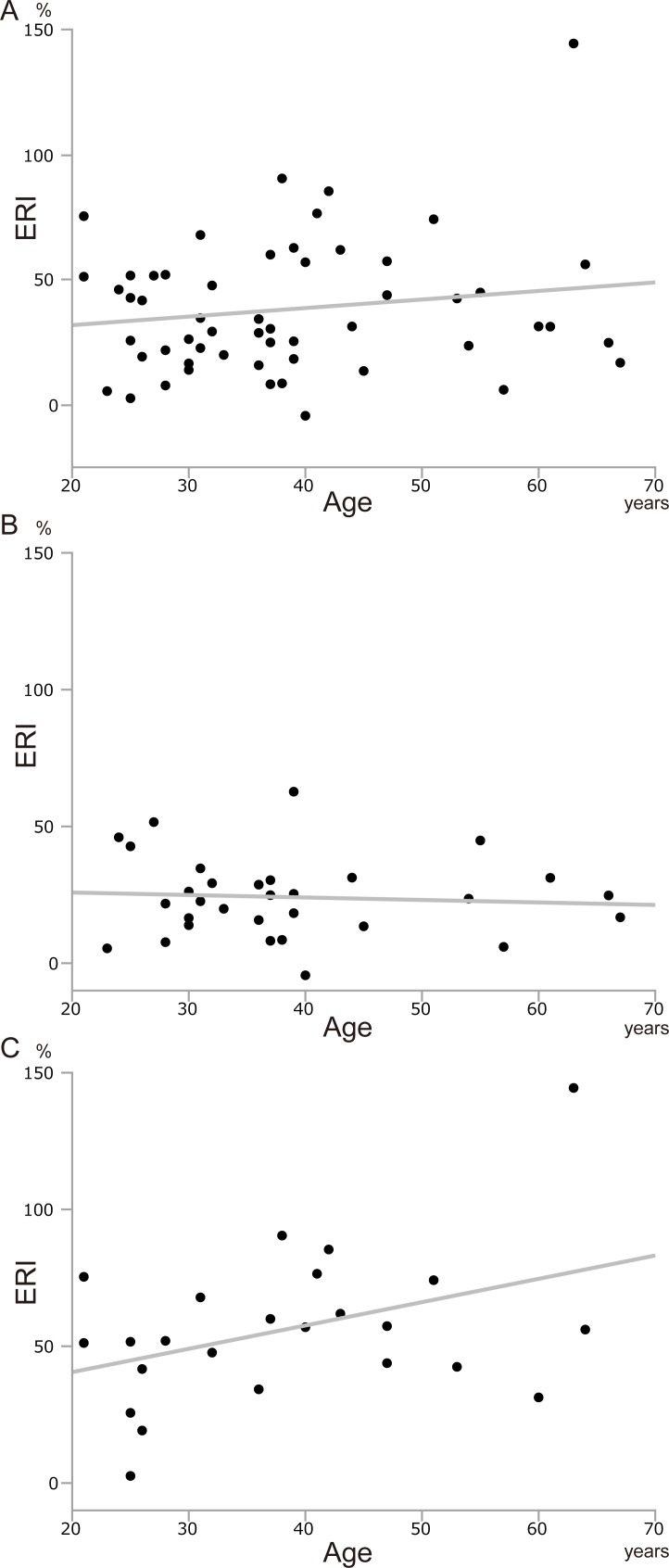
The correlation between age and liver regeneration. (A) Age and ERI in all donors (y = 0.342 x + 25.0, r^2^ = 0.025, p = 0.246). (B) Age and ERI in donors who underwent left hemihepatectomy (y = -0.091 x + 27.7, r^2^ = 0.006, p = 0.690). (C) Age and ERI in donors who underwent right hemihepatectomy (y = 0.853 x + 23.5, r^2^ = 0.159, p = 0.054). ERI; early regeneration index.

**Fig 5:**
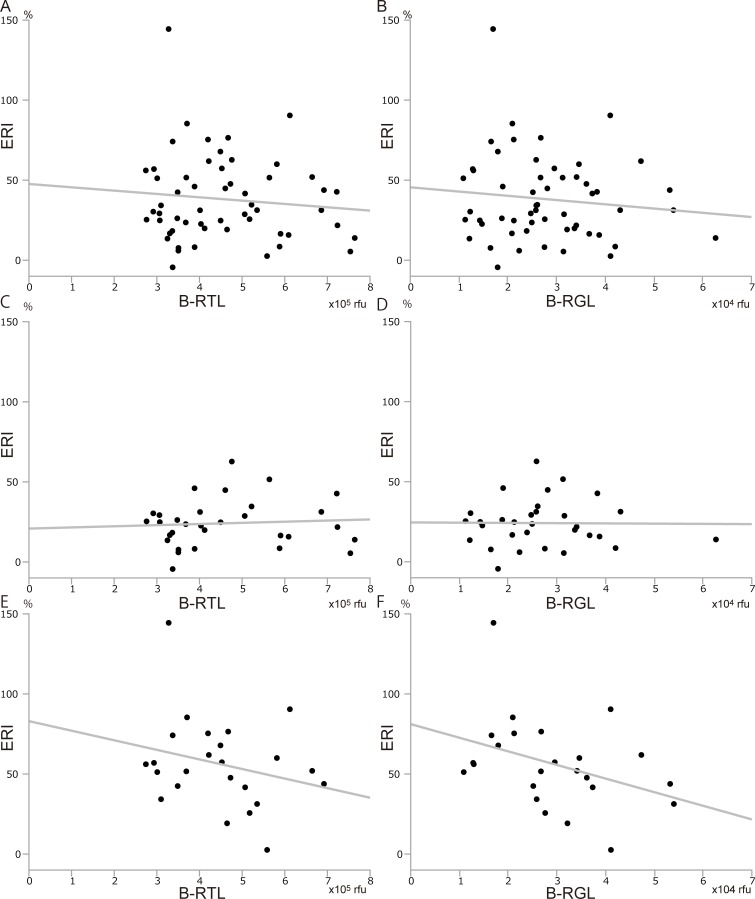
Correlation between telomere/G-tail length from blood and liver regeneration. (A) B-RTL and ERI in all donors (y = - 2.08 x + 47.6, r^2^ = 0.011, p = 0.440). (B) B-RGL and ERI in all donors (y = - 2.65 x + 45.6, r^2^ = 0.013, p = 0.403). (C) B-RTL and ERI in donors who underwent left hemihepatectomy (y = 0.71 x + 20.9, r^2^ = 0.005, p = 0.697). (D) B-RGL and ERI in donors who underwent left hemihepatectomy (y = - 0.15 x + 24.6, r^2^ < 0.001, p = 0.952). (E) B-RTL and ERI in donors who underwent right hemihepatectomy (y = - 5.98 x + 83.0, r^2^ = 0.064, p = 0.232). (F) B-RGL and ERI in donors who underwent right hemihepatectomy (y = - 8.50 x + 81.2, r^2^ = 0.138, p = 0.074). ERI; early regeneration index.

**Fig 6:**
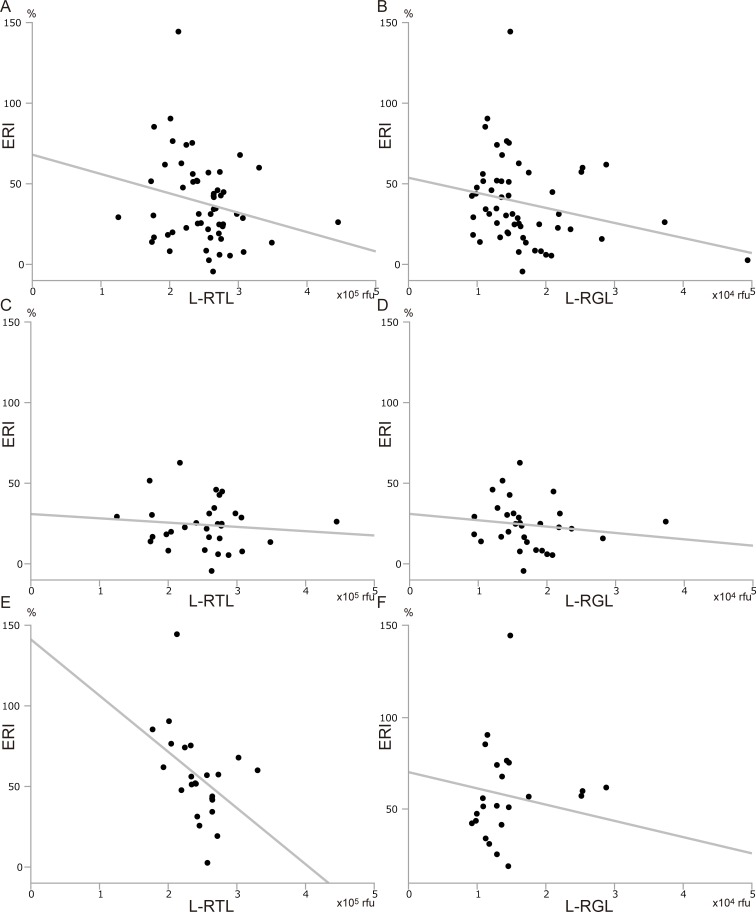
Correlation between telomere/G-tail length from liver tissue and liver regeneration. (A) L-RTL and ERI in all donors (y = - 12.0 x + 68.1, r^2^ = 0.052, p = 0.094). (B) L-RGL and ERI in all donors (y = - 9.34 x + 53.8, r^2^ = 0.062, p = 0.068). (C) L-RTL and ERI in donors who underwent left hemihepatectomy (y = - 2.66 x + 30.9, r^2^ = 0.012, p = 0.558). (D) L-RGL and ERI in donors who underwent left hemihepatectomy (y = - 3.94 x + 31.0, r^2^ = 0.022, p = 0.424). (E) L-RTL and ERI in donors who underwent right hemihepatectomy (y = - 34.9 x + 141.2, r^2^ = 0.183, p = 0.037). (F) L-RGL and ERI in donors who underwent right hemihepatectomy (y = - 8.82 x + 70.3, r^2^ = 0.076, p = 0.193). ERI; early regeneration index.

We assessed the factors which may affect postoperative liver regeneration. To investigate the differences between the regeneration rates after left and right hemihepatectomy, we examined ERI separately for left and right hemihepatectomy. Donor age was not correlated with ERI in any donor, regardless of the procedure they underwent (Fig 4A–4C). ERI was not associated with the B-RTL and B-RGL of the donor who underwent left or right hemihepatectomy (Fig 5A–5F). These results indicated that donor age and RTL/RGL from blood did not affect liver regeneration. On the other hand, the longer the L-RTL, the smaller the ERI tended to be, especially with significant difference in donor who underwent right hemihepatectomy (Fig 6A, 6C, and 6E). Similarly, the longer the L-RGL, the smaller the ERI tended to be (Fig 6B, 6D, and 6F). These results suggested that longer telomere of the liver tissue may have a negative influence on liver regeneration.

### Risk factors affecting recipient outcomes after LT

**Fig 7:**
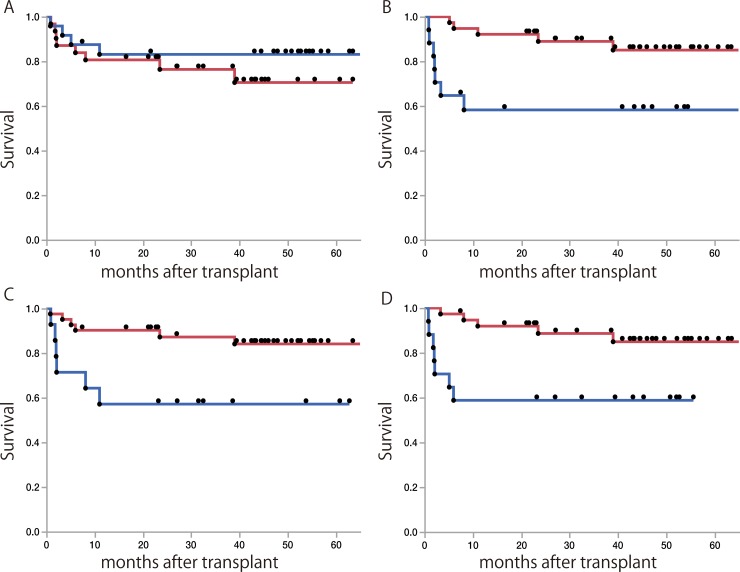
Survival curves of recipients after LDLT. (A) Comparison of the cumulative survival curves stratified with B-RTL (< 4.6 x 10^5^ rfu, red line, and ≥ 4.6 x 10^5^ rfu, blue line). (B) Comparison of the cumulative survival curves stratified with L-RTL (< 2.7 x 10^5^ rfu, red line, and ≥ 2.7 x 10^5^ rfu, blue line). (C) Comparison of the cumulative survival curves stratified with donor age (< 45 years old, red line, vs ≥ 45 years old, blue line). (D) Comparison of the cumulative survival curves stratified with MELD score (< 18, red line, and ≥ 18, blue line).

We reviewed the postoperative outcomes of recipients after LDLT (Table 3). While the survival of patients was similar between patients with shorter and longer B-RTL (p = 0.389, Fig 7A), the survival of patients with longer L-RTL was significantly inferior to that of patients with a shorter one (p = 0.007, Fig 7B). Furthermore, the survival rate of patients with older donors was significantly inferior to that of patients with younger ones (p = 0.013, Fig 7C), and the survival of patients with higher MELD scores was significantly inferior to that of patients with lower scores (p = 0.008, Fig 7D). Longer L-RTL, older donors and higher MELD scores were the independent factors affecting recipient survival after LDLT (Table 3).

**Table 3:** Prognostic factors of recipients after liver transplantation.

	univariate analysis			multivariate analysis		
	1-year survival (%)	3-year survival (%)	p value	Relative risk	*p* value	95% confidence interval
**Donor factors**						
Age, years			0.013	3.61	0.045	1.03–12.63
<45 (n = 41)	90.2	84.1				
≥45 (n = 14)	57.1	57.1				
Gender			0.955			
Male (n = 37)	81.1	81.1				
Female (n = 18)	83.3	76.4				
Type of Grafts			0.775			
Right lobe (n = 24)	83.3	78.7				
Others (n = 31)	80.7	75.6				
Diet			0.691			
Required (n = 15)	86.7	69.3				
Not required (n = 40)	80.0	80.0				
B-RTL, rfu			0.389			
<4.6 x10^5^ (n = 31)	80.7	76.4				
≥4.6 x10^5^ (n = 24)	83.1	83.1				
L-RTL, rfu			0.007	5.82	0.007	1.64–22.60
<2.7 x10^5^ (n = 38)	92.1	89.0				
≥2.7 x10^5^ (n = 17)	58.2	58.2				
B-RGL, rfu			0.836			
<2.5 x10^4^ (n = 23)	78.3	78.3				
≥2.5 x10^4^ (n = 32)	84.2	77.0				
L-RGL, rfu			0.782			
<2.0 x10^4^ (n = 43)	83.7	80.8				
≥2.0 x10^4^ (n = 12)	75.0	75.0				
**Recipient factors**						
Age, years			0.328			
<60 (n = 25)	79.8	74.5				
≥60 (n = 30)	83.3	83.3				
Gender			0.491			
Male (n = 27)	77.8	72.9				
Female (n = 28)	85.7	85.7				
MELD score			0.008	7.52	0.016	1.44–46.90
<18 (n = 38)	92.0	85.0				
≥18 (n = 17)	58.8	58.8				
Child-Pugh score			0.091	0.58	0.537	0.10–3.15
<10 (n = 33)	90.9	87.3				
≥10 (n = 22)	67.9	67.9				
HCV infection			0.408			
positive (n = 26)	76.6	71.1				
negative (n = 29)	86.2	82.1				
ABO compatibility			0.905			
identical/compatible (n = 51)	82.3	79.8				
incompatible (n = 4)	75.0	75.0				
GRWR, %			0.961			
<80 (n = 18)	83.3	76.4				
≥80 (n = 37)	81.0	77.6				
GW/SLV, %			0.384			
<40 (n = 13)	76.9	67.3				
≥40 (n = 42)	83.2	80.3				
Operation time, min			0.853			
<720 (n = 17)	76.5	76.5				
≥720 (n = 38)	84.1	80.8				
Blood loss, g			0.509			
<5000 (n = 36)	80.5	77.4				
≥5000 (n = 19)	84.2	84.2				
Ischemic time, min			0.548			
<120 (n = 27)	85.2	80.9				
≥120 (n = 28)	78.6	74.0				
Portal pressure, mmHg			0.258			
<15 (n = 34)	88.1	84.7				
≥15 (n = 21)	71.4	71.4				

MELD; Model for End-Stage Liver Disease, HCV; hepatitis C virus, GRWR; graft-to-recipient weight ratio, GW; graft weight, SLV; standard liver volume

## Discussion

### Age and LT

The impact of donor age on LT has been analyzed in several studies [1, 15]. Several reports on deceased donor liver transplantation have shown that LT performed with grafts from elderly donors had a significantly poorer graft survival than that performed with grafts from younger donors [16, 17]. Although some reports showed contradictory results, several mathematical formulas designed to predict graft outcomes, such as the donor risk index and survival outcomes following liver transplantation score, include donor age [18, 19]. Reports on LDLT have demonstrated poorer survival rates with elderly donors [20, 21]. Elderly donors have also been linked to an increased rate of biliary complications, small-for-size graft syndrome, and hepatitis C virus-related graft failure [21, 22]. In our study, donor age was identified as one of the independent factors for recipient survival after LDLT.

### Age and liver regeneration

Liver regeneration rate after hepatectomy has been shown to be inversely correlated with age [2, 23, 24]. A significant decrease in the regenerative capacity of the liver with increasing age has been reported in an animal model [25]. It is generally considered that aging negatively affects liver regeneration. However, it remains controversial whether age affects liver regeneration after hepatectomy [26]. Russolillo et al. reported that liver regeneration after portal vein occlusion was not impaired by age [27]. In our study, donor age was not correlated with early liver regeneration of donors after hepatectomy, and the mechanisms that affect liver regeneration after hepatectomy remain unclear. We hypothesized that the difference in telomere and G-tail length, which is reportedly shortened with age, impacts liver regeneration.

### Length of liver telomere/G-tail and age

Telomeres appear to be deeply involved in tissue regeneration, lifespan, and cell division [3]. They are made of double-stranded DNA containing repeat sequences of 5'-TTAGGG-3' at the ends of chromosomes. Those repeat sequences (TTAGGG) are single strands of approximately 50 to 300 bases at the furthest 3' ends (G-overhangs), called G-tails. These G-tails are normally protected by forming a loop, except when telomerase, a telomere extension enzyme, interacts with the telomere, for instance, during DNA replication [28]. Telomere length decreases as the time for cell divisions increases [3], thus according to the general theory, telomere length is inversely correlated with age [29].

In the present study, B-RTL was inversely correlated with age, consistent with previous studies. However, B-RGL, L-RTL, and L-RGL were not correlated with donor age, and L-RTL was not correlated with B-RTL. Telomere shortening in liver tissues during aging has been reported in some studies [30–32]: for example, Aikata et al. showed that telomere repeats were reduced in people with normal liver tissues by approximately 120 bp annually [30]. Takubo et al. studied telomere length in the normal liver tissue of 94 human subjects aged between 0 and 101 years old, and showed that telomere length demonstrated accelerated shortening, with a reduction of 55 bp per year [31]. Wiemann et al. showed telomere shortening in cirrhosis compared with noncirrhotic samples, independent of patients age [32]. However, it remains unclear whether L-RTL is correlated with B-RTL. The liver tissues show very little mitotic activity, indicating that there must be factors other than cell division modulating the attrition of telomeres during the aging process [3]. The kinetics of telomere shortening during aging are not linear: telomere shortening is accelerated in peripheral blood cells in young infants, reaches a plateau in older children, and slowly decreases in adulthood [3]. In addition, hepatocytes are known to stay in the G0 phase under normal conditions, retaining a very high ability of regeneration. Additionally, it has been reported that the expression and function of telomerase increase in cells when the cell cycle transitions from the S-phase to the G2-phase [33]. These reports may explain why telomere length in liver tissues is not correlated with age. In previous in vitro and in vivo experiments, the exact causal relationship between telomeres and cell aging could not be demonstrated. Specifically, it remains unclear whether the loss of cell division ability is caused by a shortening of the telomeres, or whether stress to the cells causes an apoptotic signal, such as telomere shortening [34–37]. Unlike the shortening of telomeres in somatic cells, which results in chromosome instability, shortening of the G-tail is transient depending on factors such as oxidative stress, which can be restored by environmental improvement. Thus, it is difficult to argue the relationship between telomeres and aging and life expectancy without these changing factors. Therefore, the lack of correlation between age and telomere shortening in liver tissues might open the future possibility of organ donations from elderly subjects.

### Length of liver telomere/G-tail and liver regeneration

This is the first study investigating the length of liver telomere/G-tail and liver regeneration after hepatectomy in LDLT. Several reports have investigated telomere length and telomerase activity in patients with liver disease. Aikata et al. reported that telomere repeats were shorter in the liver of patients with chronic diseases than in normal age-matched livers [30], and Hartmann et al. showed that telomerase gene mutations were present in patients with cirrhosis [38]. Telomere shortening might impair liver regeneration and accelerate cirrhosis formation [38]. Wang et al. showed in vivo accumulation of c-H2AX foci in hepatocytes in aged mice [39]. Wiemann et al. reported that telomeres were significantly shorter in cirrhosis samples than in noncirrhotic samples, independent of primary etiology and patient age [32]. Andert et al. reported that telomere length in rat hepatocytes depends on age, and animals with long telomeres had earlier and better regeneration of healthy liver tissues than rats with shorter telomeres [40]. According to these reports, longer telomere length in the donor organ tissues might provide increased liver regeneration. However, these reports showed a parallel—but not direct—association between telomere length and liver regeneration, and our results also showed no correlation between liver regeneration and telomere/G-tail length from blood. On the other hand, the longer telomere from liver tissue negatively affected liver regeneration in donor who underwent right hemihepatectomy. These results suggested that longer liver telomere may have a negative influence on liver regeneration.

Cellular senescence is considered to be a stress-response limiting the proliferation of damaged cells and leading to permanent cell-cycle arrest [41]. The telomere and telomerase systems are representative of a mediator of replicative capacity [42]. Accelerated telomere shortening has been shown to occur in conditions associated with inflammation and accelerated cell turnover [3]. A previous report showed that forced telomere elongation in cancer cells promotes their differentiation in vivo [43]. It has been reported that hepatocytes account for 64% of normal liver cells [44]. L-telomeres, which represent the telomere length of liver tissues, are clusters consisting of various cell types. Stem cells, bone marrow-derived cells, biliary duct cells, and vascular endothelial cells all have long telomeres, which are the niche of stem cells for liver regeneration [45, 46]. Conversely, in the absence of some special drugs or circumstances, the source of liver stem cells are hepatocytes, and the involvement of non-hepatocytes sources is considered to be almost negligible [47, 48]. The ploidy of eukaryotic genes can be different during the life cycle and has been reported to be dynamic[49]; therefore, hepatocytes could undergo hypertrophy and mitosis during liver regeneration. The DNA ploidy of liver cells has been reported to increase conversely (into tetraploid form) after regeneration [50]. These facts reflect that even if some hepatocytes undergo chromosome damage, some of the other cell populations can maintain the proliferative capacity by endo-replication [51].

### Length of donor liver telomere/G-tail and transplant recipient outcome

To our knowledge, this is the first study investigating the association between telomere length of donor tissues and recipient outcome in LDLT. In organ transplantation, it remains unclear whether donor tissue telomere length is associated with recipient survival. A previous study showed that longer donor telomere length is associated with improved recipient survival among hematopoietic cell transplant recipients with aplastic anemia [52]. In contrast, Courtwright et al. reported that neither donor or recipient telomere length were significantly associated with survival after lung transplantation [53]. Our study showed that longer telomere length of donor liver tissues was associated with lower recipient survival. A previous study examined telomere length in tumor and adjacent non-tumor tissues from 126 US patients with hepatocellular carcinoma, and showed no correlation between survival and telomere length in both tumor and adjacent non-tumor tissues [54]. Therefore, the question of whether telomere length in liver tissues is associated with patient survival remains controversial.

This study has several limitations. First, although this is the first study to investigate specifically on telomere and G-tail length from healthy donor liver tissue, we had a relatively small cohort with a limited follow-up period, and the age of donors included was limited to less than 65 years only. Second, a cell cluster of whole liver tissue used in our study included not only hepatocytes, but also other cells including cholangiocytes, and sinusoidal cells. Third, we did not assess telomerase activity or the telomere/G-tail in transplant recipients. Fourth, we assessed telomere length by HPA assay, as a flow-based assay. Telomere length is measured as fluorescence intensity relative to internal control, not as an absolute length [4].

## Conclusion

Telomere shortening in healthy liver tissue was not correlated with age, whereas longer liver telomeres negatively impact donor liver regeneration and recipient survival after LDLT. These results can direct future studies and investigations on telomere shortening in the clinical and experimental transplant setting.

## Supporting information

S1 Table (XLSX)
